# Intestinal Inflammation Responds to Microbial Tissue Load Independent of Pathogen/Non-Pathogen Discrimination

**DOI:** 10.1371/journal.pone.0035992

**Published:** 2012-05-07

**Authors:** Yvonne Willer, Beatrice Müller, Dirk Bumann

**Affiliations:** 1 Junior Group Mucosal Infections, Hannover Medical School, Hannover, Germany; 2 Department of Molecular Biology, Max-Planck-Institute for Infection Biology, Berlin, Germany; 3 Infection Biology, Biozentrum, University of Basel, Basel, Switzerland; French National Centre for Scientific Research - Université Aix-Marseille, France

## Abstract

The intestinal immune system mounts inflammatory responses to pathogens but tolerates harmless commensal microbiota. Various mechanisms for pathogen/non-pathogen discrimination have been proposed but their general relevance for inflammation control is unclear. Here, we compared intestinal responses to pathogenic *Salmonella* and non-pathogenic *E. coli*. Both microbes entered intestinal Peyer’s patches and, surprisingly, induced qualitatively and quantitatively similar initial inflammatory responses revealing a striking discrimination failure. Diverging inflammatory responses only occurred when *Salmonella* subsequently proliferated and induced escalating neutrophil infiltration, while harmless *E. coli* was rapidly cleared from the tissue and inflammation resolved. Transient intestinal inflammation induced by harmless *E. coli* tolerized against subsequent exposure thereby preventing chronic inflammation during repeated exposure. These data revealed a striking failure of the intestinal immune system to discriminate pathogens from harmless microbes based on distinct molecular signatures. Instead, appropriate intestinal responses to gut microbiota might be ensured by immediate inflammatory responses to any rise in microbial tissue loads, and desensitization after bacterial clearance.

## Introduction

The intestinal mucosa is a major site of pathogen entry. To fight infection, the intestinal innate immune system detects pathogens and tries to control them with immediate inflammatory responses. Important receptors for pathogen detection include toll-like receptors (TLRs) and NOD-like receptors that recognize bacterial components such as lipopolysaccharide, peptidoglycan, and flagella [1]. However, similar microbe-associated molecular patterns (MAMPs) are also present on harmless or even beneficial bacteria that inhabit the gut in vast numbers [2], [3]. In fact, harmless bacteria are also recognized by the intestinal immune system through TLRs but in this case, signaling supports epithelial integrity and prevents damage-induced inflammation [4], [5]. These similar recognition pathways for both pathogens and harmless microbiota raise the question how appropriate divergent inflammatory responses can be ensured.

Various molecular mechanisms have been proposed to discriminate pathogens from harmless gut bacteria including (i) poorly stimulatory microbial-associated molecular patterns (MAMP) in commensals but potent MAMPs in pathogens, (ii) “symbiont-associated molecular patterns” (SAMPs) that suppress inflammation [6], (iii) pathogens producing distinct MAMP combinations related to their growth characteristics, (iv) pathogen interaction with epithelial surface receptors and introduction of MAMPs into host cells, and (v) pathogen factors that cause specific tissue damage generating “danger signals”. Experimental evidence supports each mechanism but also reveals their limitations [7], [8]. In particular, it is unclear which mechanisms ensure appropriate responses to diverse pathogens with widely differing virulence mechanisms, to commensals that turn into opportunistic pathogens, and to diverse harmless food-contaminating microbes.

In this study, we compared intestinal inflammatory responses to pathogenic *Salmonella* and non-pathogenic *E. coli* in murine intestinal Peyer’s patches, and characterized underlying mechanisms using mouse mutants and various *Salmonella* strains. The results suggested surprising similar initial inflammatory responses to both pathogenic/avirulent *Salmonella* and harmless *E. coli* indicating a striking discrimination failure. However, appropriate responses were still possible since inflammation depended on bacterial tissue load. *Salmonella,* which survived and proliferated in Peyer’s patches, thus induced escalating inflammation, while harmless *E. coli* was rapidly cleared and inflammation resolved.

## Results

### Inflammatory Responses to *Salmonella*

**Figure 1:**
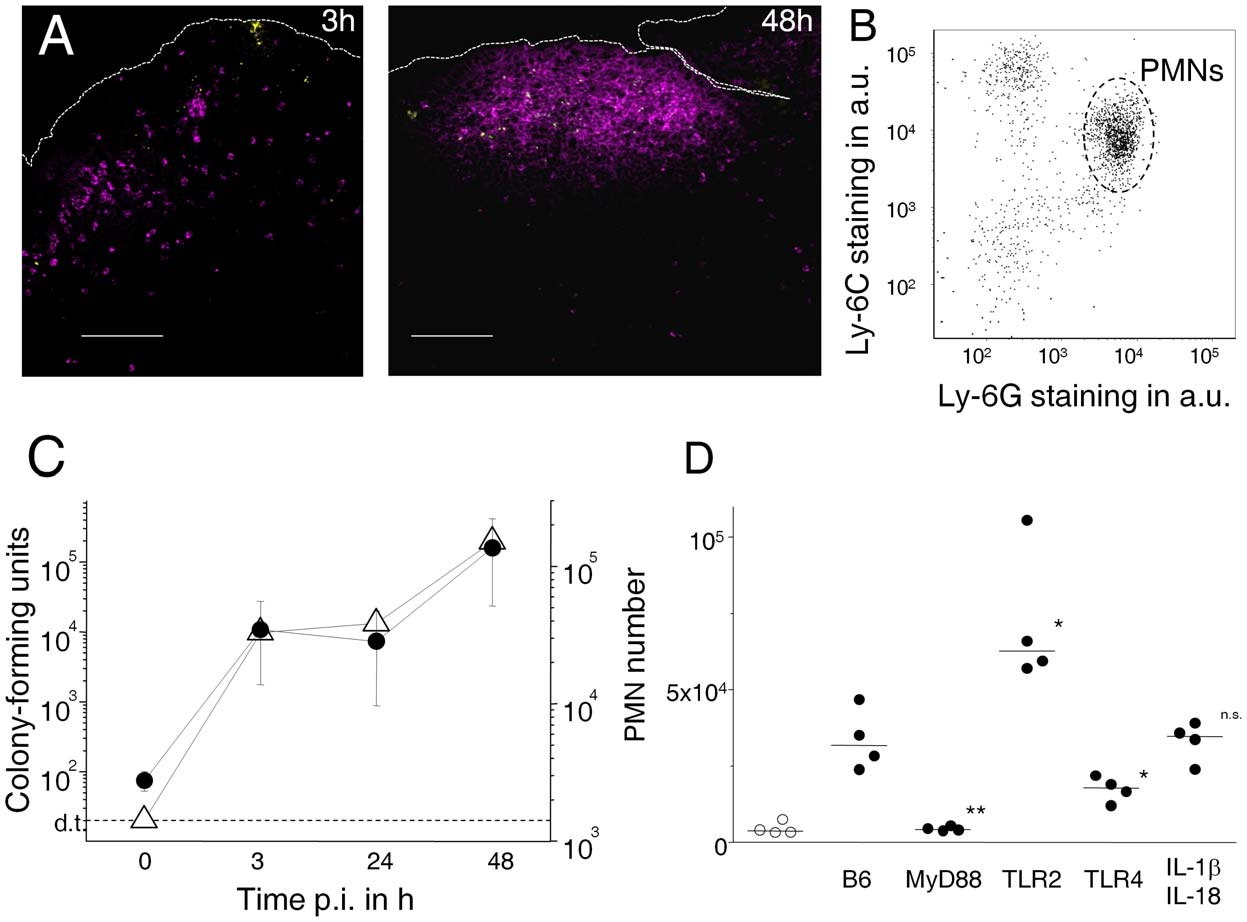
*Salmonella* infection induces intestinal inflammation. **A**) Polymorphonuclear neutrophils (PMN, magenta) accumulate close to *Salmonella* (yellow) in the dome area of murine Peyer’s patches 3 h or 48 h after intragastric infection. The scale bar represents 100 µm. Peyer’s patches of naïve mice contained only few neutrophils (data not shown). **B**) Phenotype of infiltrating CD11b^+^ host cells 3 h post infection. The majority of cells represent PMN’s (Ly-6G^hi^ Ly-6C^mo^), some other cells represent mobile monocytes (Ly-6G^lo^ Ly-6C^hi^). **C**) Colonization levels of *Salmonella* (open triangles) and PMN infiltration (filled circles) in Peyer’s patches at various time intervals post infection (d.t., detection threshold for plating). The data might overestimate actual bacterial tissue loads due to potential contamination with residual luminal *Salmonella* especially at early time points after oral administration. Means and standard deviations are shown for groups of three to four mice. Similar data were obtained in two independent experiments. **D**) PMN infiltration 3 h post infection in wildtype C57BL/6 mice (B6) and mutant mice with various defects in innate immunity (filled circles). Data for sham-infected B6 are also shown (open circles). Significance of differences to infected B6 was determined using t-test on log-transformed data (**, *P*<0.01; *, *P*<0.05; n.s., *P*>0.05). Similar data were obtained in three independent experiments.

We investigated murine intestinal inflammatory responses to the model pathogen *Salmonella enterica* serovar Typhimurium. We initially used high doses of 10^11^ CFU that have previously been used to study intestinal immune responses to *Salmonella* [9], [10], [11], [12], and reflect typical bacterial numbers in heavily contaminated food. Three hours after oral administration of *Salmonella*, *Salmonella* were detected in dome areas of intestinal Peyer’s patches (gut-associated lymphoid tissues) (Fig. 1A) as observed previously [9]. At this early time point post infection, activated polymorphonuclear neutrophils (PMN; Ly-6C^me^ Ly-6G^hi^ CD11b^hi^) and some mobile monocyte precursors (Ly-6C^hi^ Ly-6G^lo^ CD11b^hi^) had already infiltrated infected dome areas (Fig. 1A,B,C; Fig. S1) as previously observed [13], [14], [15], [16]. Within 48 h, *Salmonella* proliferated to high loads in Peyer’s patches, and neutrophils accumulated in large numbers. We did not observe substantial neutrophil infiltration in the villous lamina propria suggesting that inflammation predominantly localized to Peyer’s patches.

*Salmonella* triggered initial neutrophil infiltration through activation of MyD88 with some involvement of TLR4 but not IL-1β or IL-18 (Fig. 1D) in agreement with previous observations [16], [17], [18]. Interestingly, mice lacking TLR2 had even higher neutrophil counts compared to congenic wildtype mice. This suggested a potential anti-inflammatory role of TLR2 in the intestine in agreement with earlier observations [19], [20]. At later time points, substantial neutrophil infiltration was observed even in mice deficient in MyD88 or TLR4 (Fig. S2) suggesting that multiple partially redundant pathways triggered neutrophil infiltration at high *Salmonella* loads in agreement with previous data for systemic salmonellosis [17]. Similar inflammatory responses were also observed in another inbred mouse line (BALB/c, data not shown).

### Inflammatory Responses to Harmless *E. coli*

**Figure 2:**
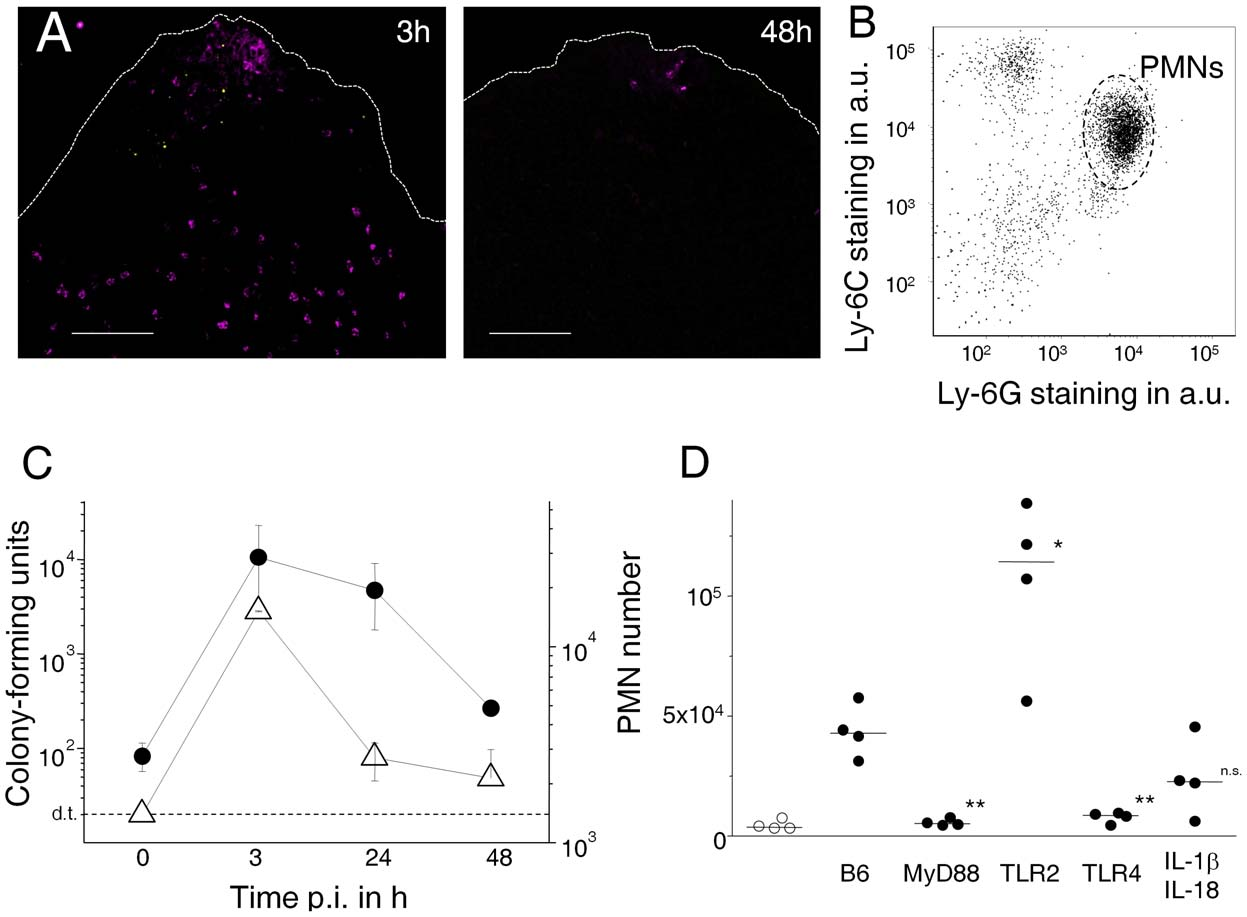
Probiotic *E. coli* Nissle (EcN) initially induces intestinal inflammation that is qualitatively and quantitatively similar to early responses to *Salmonella*. **A**) Polymorphonuclear neutrophils (PMN, magenta) accumulate close to EcN (yellow) in the dome area of murine Peyer’s patches 3 h after intragastric EcN administration. Bacteria are cleared and inflammation resolves within 48 h after administration. The scale bar represents 100 µm. Similar results were obtained for 12 mice in three independent experiments. **B**) Phenotype of infiltrating CD11b^+^ host cells 3 h post infection. The majority of cells represent PMN’s (Ly-6G^hi^ Ly-6C^mo^), some other cells represent mobile monocytes (Ly-6G^lo^ Ly-6C^hi^). **C**) Colonization levels of EcN (open triangles) and PMN infiltration (filled circles) in Peyer’s patches at various time intervals post infection (d.t., detection threshold for plating). The data might overestimate actual bacterial tissue loads due to potential contamination with residual luminal EcN especially at early time points after oral administration. Means and standard deviations are shown for groups of three to four mice. Similar data were obtained in four independent experiments. **D**) PMN infiltration 3 h post EcN administration in wildtype C57BL/6 mice (B6) and mutant mice with various defects in innate immunity. Data for sham-infected B6 are also shown (open circles). Significance of differences to infected B6 was determined using t-test on log-transformed data (**, *P*<0.01; *, *P*<0.05; n.s., *P*>0.05). Similar data were obtained in two independent experiments.

We compared this well-characterized inflammatory response to pathogenic *Salmonella* with responses to non-pathogenic *E. coli* Nissle 1917 (EcN). EcN is a probiotic bacterium that can prevent or ameliorate chronic intestinal inflammation in animal models and human patients [21]. Within three hours after oral administration, detectable numbers of EcN reached Peyer’s patches dome areas (Fig. 2A,C) consistent with previous observations of intestinal particle uptake [22]. Surprisingly, neutrophils and some mobile monocytes had infiltrated Peyer’s patch dome areas at the same time in numbers and proportions (Fig. 2A,B,C; Fig. S1) similar to what we had observed for pathogenic *Salmonella* (Fig. 1; Fig. S1). Again, we did not observe substantial infiltration of the villous lamina propria.

The early transient inflammatory response to EcN depended on TLR4 and MyD88 but not Il-1β or IL-18 (Fig. 2D). Again, TLR2-deficient mice had even higher responses compared to wildtype mice. Interestingly, initial inflammatory responses to both bacteria had similar dose-dependencies (Fig. S3). In contrast to *Salmonella*, however, EcN was subsequently cleared and inflammation largely resolved within 48 h (Fig. 2A,C). Similar inflammatory responses were also observed in another inbred mouse line (BALB/c, data not shown).

We also tested inflammatory responses to an avirulent *E. coli* E2 that we had isolated from normal murine microbiata [23]. Again, high doses induced substantial neutrophil infiltration (Fig. S4A).

These surprising data revealed qualitatively and quantitatively similar initial inflammatory responses to *Salmonella* and probiotic EcN suggesting a striking failure to discriminate pathogenic from harmless microbes.

### Impact of *Salmonella* Virulence Factors on Inflammatory Responses

**Figure 3:**
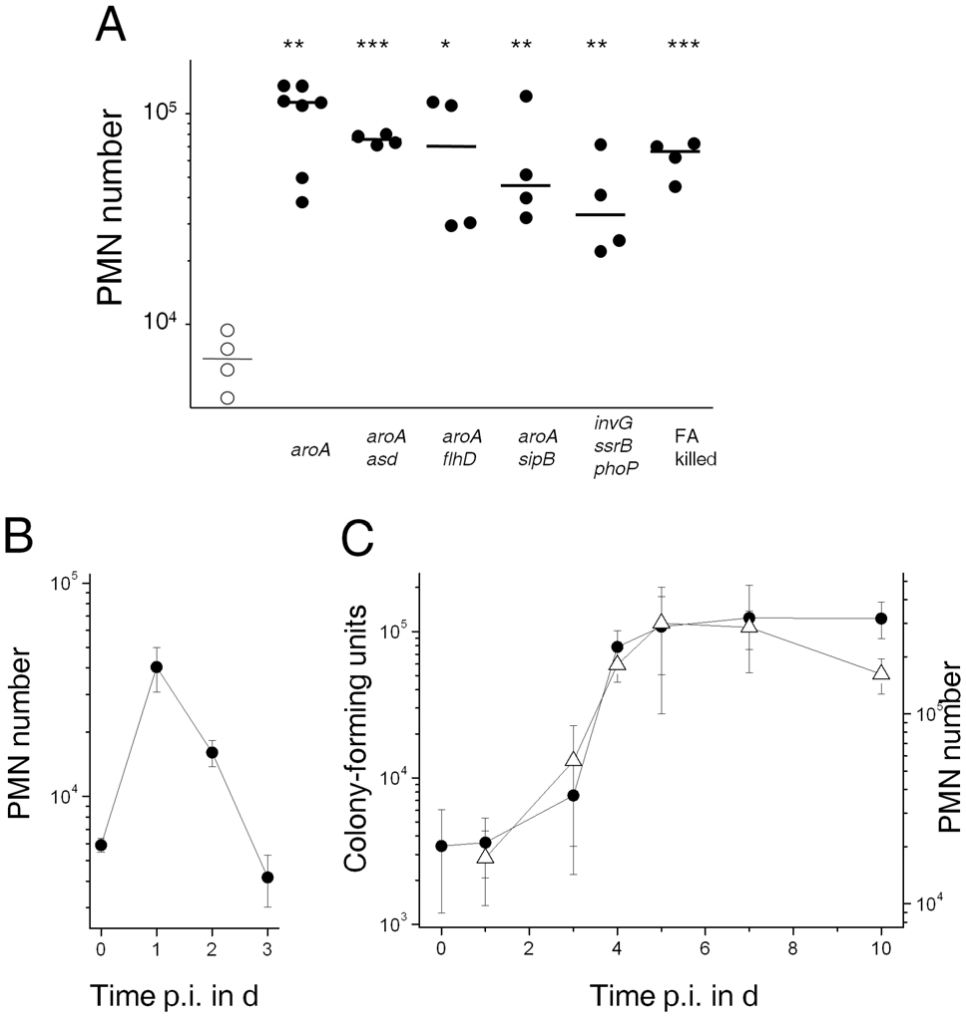
Initial inflammatory responses to *Salmonella* are independent of specific virulence factors. **A**) PMN infiltration 3 h post administration of various live *Salmonella* mutants in BALB/c mice. Data for formalin (FA) killed wildtype *Salmonella* are also shown. Significance of differences to baseline levels (open circles) was determined using t-test on log-transformed data (***, *P*<0.001; **, *P*<0.01; *, *P*<0.05). **B**) PMN infiltration (filled circles) in Peyer’s patches of C57BL/6 mice at various time intervals post infection with avirulent *Salmonella aroA asd*. Means and standard deviations are shown for groups of three to four mice. No colony-forming units were recovered from day one post infection consistent with rapid in vivo lysis of this cell wall-deficient strain. Similar data were obtained in an independent experiment. **C**) Colonization levels of attenuated *Salmonella aroA* (open triangles) and PMN infiltration (filled circles) in Peyer’s patches at various time intervals post infection in BALB/c mice. Means and standard errors are shown for groups of six to eight mice from two independent experiments.

Such similar responses to virulent *Salmonella* and harmless EcN were unexpected since *Salmonella* but not EcN have numerous virulence factors that subvert host responses, cause massive tissue damage, and trigger rapid inflammatory responses in in vitro cell culture experiments. To determine the in vivo impact of some of these virulence factors, we compared inflammatory responses to defined isogenic *Salmonella* mutants (Fig. 3A). Interestingly, all tested *Salmonella* mutants including completely avirulent *Salmonella invG ssrB phoP*, in which all three major virulence systems were inactive, induced substantial neutrophil infiltration at 3 h post infection. Avirulent *Salmonella aroA asd* that lyzed spontaneously because of a defective cell-wall, and even formalin-fixed *Salmonella* also elicited substantial neutrophil infiltration in Peyer’s patches suggesting that in vivo activities were dispensable to elicit initial inflammatory responses. In contrast to wildtype *Salmonella*, however, completely avirulent mutants such *Salmonella aroA asd* were rapidly cleared from Peyer’s patches and inflammation resolved (Fig. 3B). For comparison, we also followed responses to moderately attenuated *Salmonella aroA* (Fig. 3C). Following infection with a reduced dose (10^9^ CFU), there was little initial neutrophil accumulation. However, *Salmonella* slowly proliferated to high tissue loads and this was associated with a parallel neutrophil accumulation as observed for wildtype *Salmonella* at a faster time scale (compare Fig. 1C). Together, these data suggested that the intestinal immune system responded to *Salmonella* based on tissue loads regardless of specific virulence properties.

### Rapid Tolerance by Harmless Bacteria

The inflammatory response to harmless EcN was surprising since control mice that were continuously exposed to harmless endogenous microbiota, had low baseline neutrophil numbers in their Peyer’s patches (Figs. 1, 2). However, such inflammatory responses to EcN required rather high oral doses (Fig. S3) that provided strong bacterial stimulation compared to normal exposure levels.

**Figure 4:**
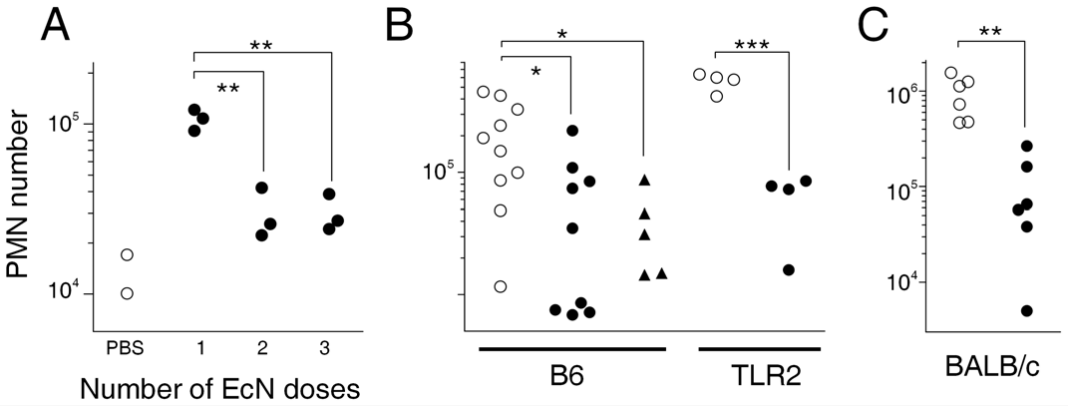
Administration of harmless bacteria induces rapid tolerance to non-pathogenic and pathogenic gut bacteria. **A**) Neutrophil infiltration in murine Peyer’s patches 3 h after the last of one, two, or three daily doses of probiotic *EcN* to BALB/c mice. Neutrophils numbers in control mice that received only PBS are also shown. Similar data were obtained in two independent experiments. **B**) Neutrophil accumulation in Peyer’s patches two days after oral infection with pathogenic *Salmonella* in wildtype C57BL/6 mice (B6) and mutant mice deficient in TLR2. One day before *Salmonella* infection, mice received PBS (open circles), or a single dose of live (filled circles) or formalin-fixed (filled triangles) *EcN*. Similar data were obtained in one independent experiment. **C**) Neutrophil accumulation in Peyer’s patches two days after oral infection with pathogenic *Salmonella* in BALB/c mice. One day before *Salmonella* infection, mice received PBS (open circles), or a single dose of avirulent live *Salmonella aroA asd* (filled circles). Pooled data from two independent experiments are shown. Significance of differences between groups was determined using t-test (***, *P*<0.001; **, *P*<0.01; *, *P*<0.05).

To determine consequences of repeated exposure to such high bacterial doses, we administered multiple daily doses of ca. 5×10^10^ CFU *E. coli* Nissle 1917. Interestingly, only the first dose elicited strong immediate neutrophil infiltration whereas subsequent doses were largely ineffective (Fig. 4A). A sudden exposure to harmless bacteria thus triggered transient inflammation but subsequently induced rapid desensitization to the new exposure level without further inflammation. Similar tolerance induction has previously been observed in initially germ-free mice during gut colonization with a complex microflora [24], [25], and during LPS exposure immediately after birth [26]. Systemic TLR4-mediated inflammatory responses to LPS are also subject to rapid tolerance [27].

Interestingly, a single high dose of live or formalin-fixed EcN, or *E. coli* E2 isolated from normal murine microbiota [23], even suppressed subsequent inflammatory responses to virulent *Salmonella* (Fig. 4B; Fig. S4B). A single high dose of avirulent *Salmonella aroA asd* had the same effect (Fig. 4C). This similar cross-tolerance/tolerance again supported the lack of fundamentally distinct recognition pathways for pathogenic/non-pathogenic microbes. It is important to note that this tolerance required administration of high bacterial doses that provided strong stimulation compared to basal exposure to normal commensal microbiota loads.

It would be interesting to determine if pathogenic bacteria could also induce tolerance to subsequent bacterial exposure. However, the strong long-lasting inflammation during infection with pathogenic *Salmonella* presented experimental challenges to detect responses to additional bacterial doses.

Triggering of TLR2 signaling has been reported to be essential for anti-inflammatory in vitro activities of EcN [28] but in vivo, EcN induced cross-tolerance to virulent *Salmonella* independently of TLR2 (Fig. 4C). Instead, the difference in PMN accumulation in tolerized/untreated TLR2-deficient mice was even higher partially due to their hyperresponsiveness against intestinal bacterial exposure as observed in our other experiments (Fig. 1D, 2D).

The ability of EcN to suppress inflammatory responses to subsequent microbial exposure could contribute to EcN’s well-documented long-term anti-inflammatory activity [21]. The fact that formalin- inactivated EcN was as effective as live EcN (Fig. 4B) indicated that EcN in vivo activities might be dispensable for its probiotic action. This could offer practical advantages for manufacturing and storage, and permit safe high doses in patients with chronic inflammatory bowel disease, that in turn might increase treatment efficacy. Future work is required to clarify this issue.

## Discussion

The intestinal immune system fights against pathogens but tolerates vast commensal microbiota. It is possible that fundamentally different molecular recognition pathways are involved in this crucial discrimination capability. However, our data showed surprising qualitatively and quantitatively similar inflammatory responses to pathogenic *Salmonella* and harmless *E. coli* indicating a striking unexpected initial failure of the intestinal innate immune system to discriminate pathogens from harmless bacteria. On the other hand, initial inflammation was strongly dependent on the bacterial inoculum dose (Fig. S3), and subsequent inflammation closely correlated with the development of bacterial tissue loads suggesting that the amount of bacterial components present in host tissue rather than a specific molecular signature regulated inflammation.

These data suggested a model in which simple monitoring of common microbial components using a limited number of common microbial pattern recognition receptors might ensure appropriate intestinal responses to diverse gut microbes even without pathogen/non-pathogen discrimination. According to this model, any increase in microbial tissue load would represent a potential assault by invading virulent microbes that mandates immediate inflammatory responses to control infection; in contrast, decreasing microbial tissue loads could indicate clearance of harmless microbes or successful defeat of pathogens, and thus permit to terminate inflammation. When new environmental conditions increase exposure to harmless commensals, transient inflammation may occur, but this would be rapidly followed by desensitization to subsequent exposure thereby preserving intestinal homeostasis.

This model of dynamic monitoring of common microbial components could provide a simple generic solution even for particularly challenging microbial discrimination tasks. In particular, a wide range of pathogens with many diverse virulence mechanisms would be correctly identified as a potential threat based on their ability to proliferate in host tissues with no need to recognize each unique virulence mechanism with specific receptors. Similarly, commensals that can turn into opportunistic pathogens [29] would be differentially detected based on their actual current virulence potential despite similar molecular composition in the commensal and pathogenic states. Finally, diverse food-contaminating microbes might lack anti-inflammatory mechanisms to support co-existence with mammalian intestines, but intestinal tolerance would still be rapidly achieved through desensitization after exposure.

Many additional mechanisms are likely to modulate intestinal inflammation in specific microbe-host interactions [7], [8]. Future work might reveal how generic and specific recognition and response patterns interact to ensure intestinal homeostasis in the presence of diverse gut microbiota.

## Materials and Methods

### Bacterial Strains and Growth Conditions

*Salmonella* strains used in this study were derivatives of *Salmonella enterica* serovar Typhimurium SL1344 *hisG rpsL xyl* [30]. *Salmonella* mutants with defined gene deletions were obtained using the Lambda phage red recombinase method [31]. *Salmonella* was grown in Lennox LB medium containing 90 µg ml^−1^ streptomycin, 100 µg ml^−1^ ampicillin, 20 µg ml^−1^ chloramphenicol, and/or 30 µg ml^−1^ kanamycin. *Escherichia coli* Nissle 1917 (O6:K5:H1) *hly* ^−^, mcm^+^ was obtained from a local pharmacy (Mutaflor™) and grown in Lennox LB medium. A spontaneous streptomycin mutant was selected and used for mouse experiments. In addition, an avirulent *E. coli* strain that we had isolated from mouse feces [23] was also used. For intragastric infection, *Salmonella* or *E. coli* late log phase cultures were resuspended in PBS. Some bacterial suspensions were fixed with 2% formalin in PBS on ice for 1 h followed by two washes with PBS.

### Mouse Infections

All animals were handled in strict accordance with good animal practice and all animal work was approved by local animal care and use committee (license 04/862 Niedersächsisches Landesamt für Verbraucherschutz und Lebensmittelsicherheit). Eight to 12 weeks old female C57BL/6 mice, congenic gene-deficient mice female mice, or female BALB/c mice were intragastrically infected with about 5×10^10^ *E. coli* Nissle 1917, or 5×10^10^ *Salmonella* using a round-tip stainless steel needle. At various time intervals after infection Peyer’s patches were prepared. After mucus was removed using Kim-Wipes, Peyer’s patches were thoroughly washed in PBS, homogenized between the sanded ends of microscopy slides, mixed with Triton X-100 (final concentration 0.17%), and plated on LB media containing 90 µg ml^−1^ streptomycin that effectively suppressed endogenous flora.

### Immunohistochemistry

Peyer’s patches were embedded in Tissue-Tek (Sakura) and frozen on dry ice. Cryotome sections (8 µm) were blocked for 30 min (Molecular Probes blocking solution), and incubated with primary antibodies (rabbit polyclonal anti-*Salmonella*, Sifin; rabbit polyclonal anti LPS-O6 kindly provided by A. Fruth; anti-Ly-6G-FITC, BD Biosciences) followed by secondary antibodies (anti-rabbit Alexa647, Molecular Probes; anti-FITC Alexa488, Molecular Probes). Sections were examined with a confocal microscopy (Leica SP5).

### Flow Cytometry

Peyer’s patches were homogenized between the sanded ends of microscope slides to obtain single cell suspensions. Cells were stained in PBS containing 2% FCS and 2% mouse serum using monoclonal antibodies anti-CD11b-PE, anti-Ly-6C-biotin, and anti-Ly-6G-FITC followed by strepatavidin-APC (all from BD Biosciences). Stained cells were analyzed using flow cytometry (Calibur or CantoII, BD Biosciences). PMN were identified as CD11b^hi^ Ly-6G^hi^ Ly-6C^me^.

## Supporting Information

Figure S1 Infiltration of mobile monocytes (Ly-6G^lo^ Ly-6C^hi^) in Peyer’s patches after administration of virulent *Salmonella* (filled circles) or probiotic *E. coli* Nissle (EcN, open circles). Means and standard deviations are shown for groups of three to four mice (same experimental groups as shown in Figs. 1 and 2).
(TIF)

Figure S2 Neutrophil infiltration of Peyer’s patches at high *Salmonella* loads in wildtype (B6) and mutant mice with various defects in innate immunity. Infection kinetics differed between the various mouse strains. We therefore compared highly infected mice (*Salmonella* loads between 50.000 to 250.000 CFU).
(TIF)

Figure S3 Immediate neutrophil infiltration 3 h after oral administration of different doses of *Salmonella aroA* (filled circles) or *E. coli* Nissle 1917 (open circles). Means and standard errors for groups of three to five individuals from two independent experiments are shown.
(TIF)

Figure S4 Neutrophil infiltration and colonization of Peyer’s patches after administration of a murine *E. coli* strain. **A**) Colonization levels of *E. coli* E2 (open triangles) and PMN infiltration (filled circles) in Peyer’s patches at various time intervals post infection (d.t., detection threshold for plating). The data might overestimate actual bacterial tissue loads due to potential contamination with residual luminal EcN especially at early time points after oral administration. Means and standard deviations are shown for groups of three mice. **B**) Neutrophil accumulation in Peyer’s patches two days after oral infection with pathogenic *Salmonella* in BALB/c mice. One day before *Salmonella* infection, mice received PBS (open circles), or a single dose of murine *E. coli* E2 (filled circles). Significance of differences between groups was determined using t-test (*, *P*<0.05).
(TIF)
