# Supplementary figures and images for: Intestinal Inflammation Responds to Microbial Tissue Load Independent of Pathogen/Non-Pathogen Discrimination
Source: PLoS One. 2012 May 7;7(5):e35992. doi: 10.1371/journal.pone.0035992 (PMC3346762; doi:10.1371/journal.pone.0035992)

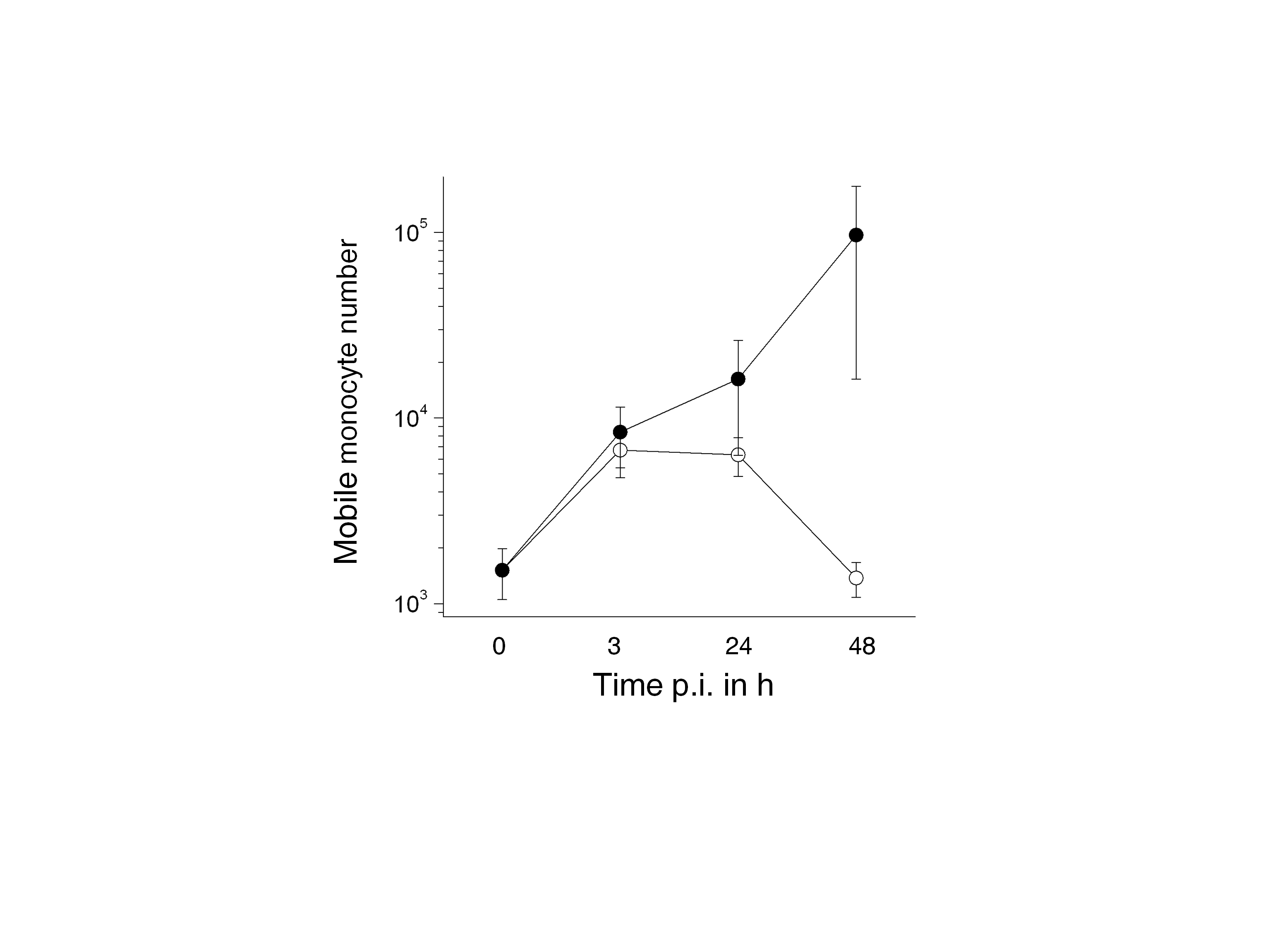

Supplement: Figure S1 — Infiltration of mobile monocytes (Ly-6Glo Ly-6Chi) in Peyer’s patches after administration of virulent Salmonella (filled circles) or probiotic E. coli Nissle (EcN, open circles). Means and standard deviations are shown for groups of three to four mice (same experimental groups as shown in Figs. 1 and 2). (TIF) [file pone.0035992.s001.tif]

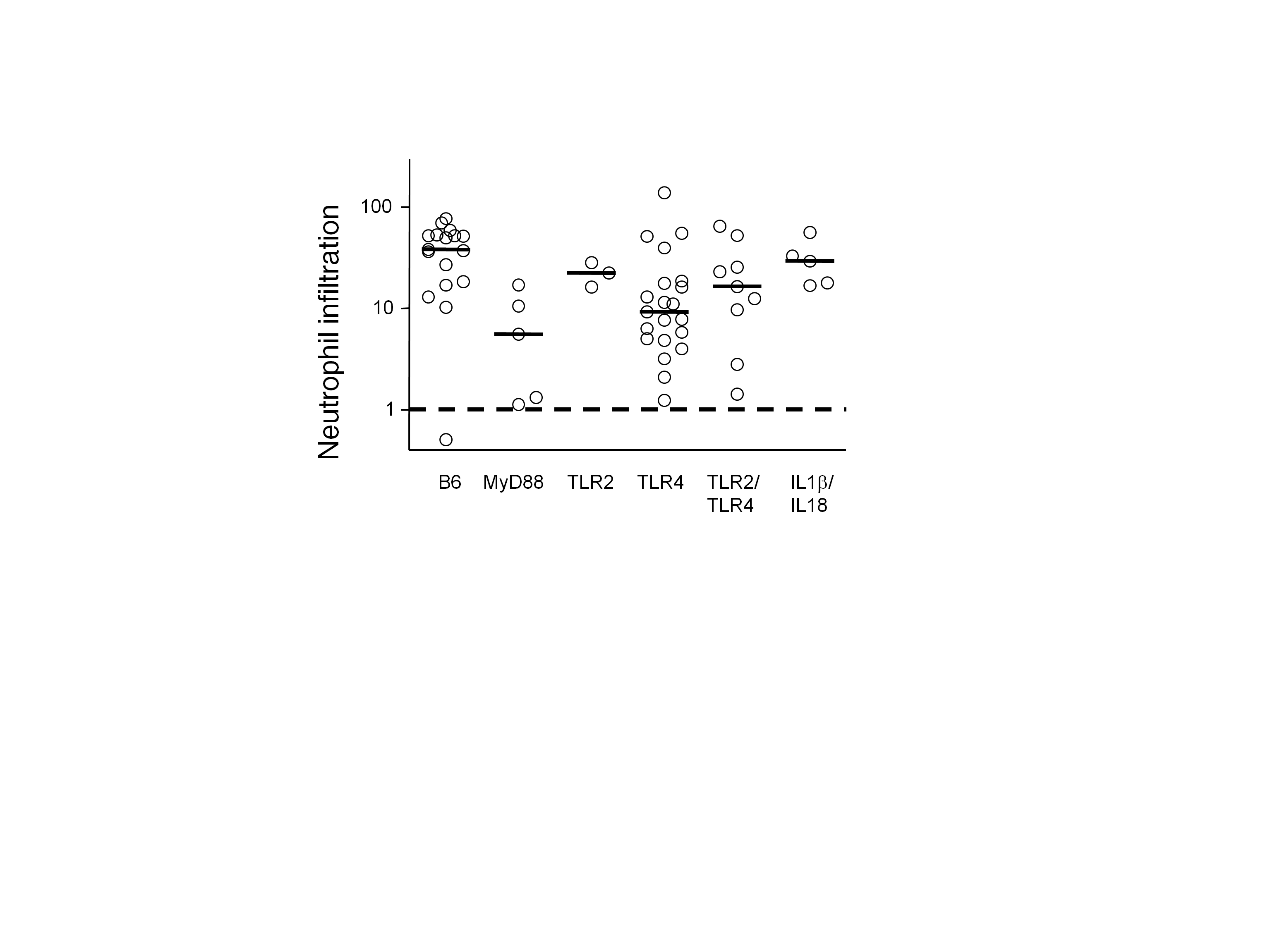

Supplement: Figure S2 — Neutrophil infiltration of Peyer’s patches at high Salmonella loads in wildtype (B6) and mutant mice with various defects in innate immunity. Infection kinetics differed between the various mouse strains. We therefore compared highly infected mice (Salmonella loads between 50.000 to 250.000 CFU). (TIF) [file pone.0035992.s002.tif]

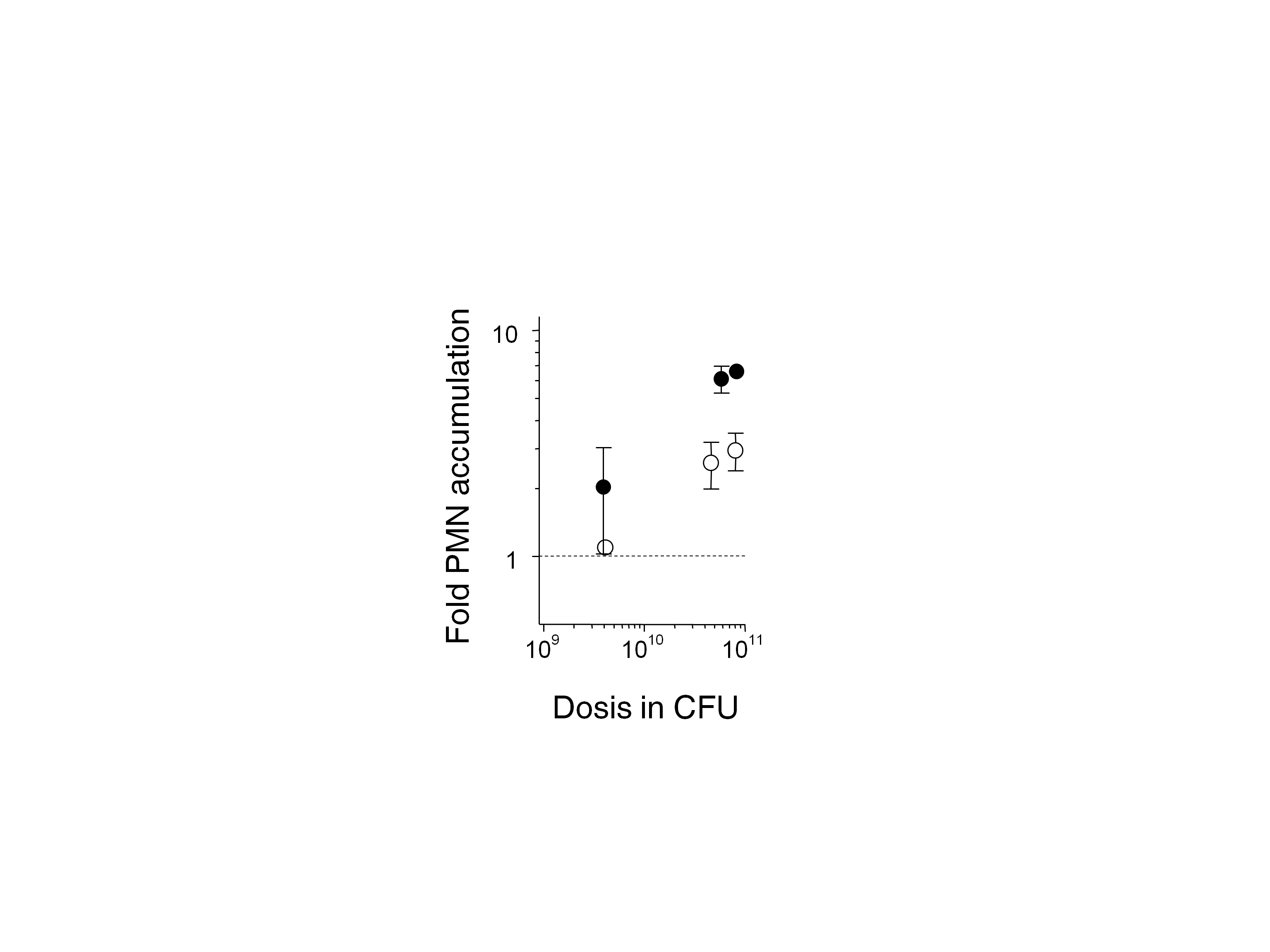

Supplement: Figure S3 — Immediate neutrophil infiltration 3 h after oral administration of different doses of Salmonella aroA (filled circles) or E. coli Nissle 1917 (open circles). Means and standard errors for groups of three to five individuals from two independent experiments are shown. (TIF) [file pone.0035992.s003.tif]

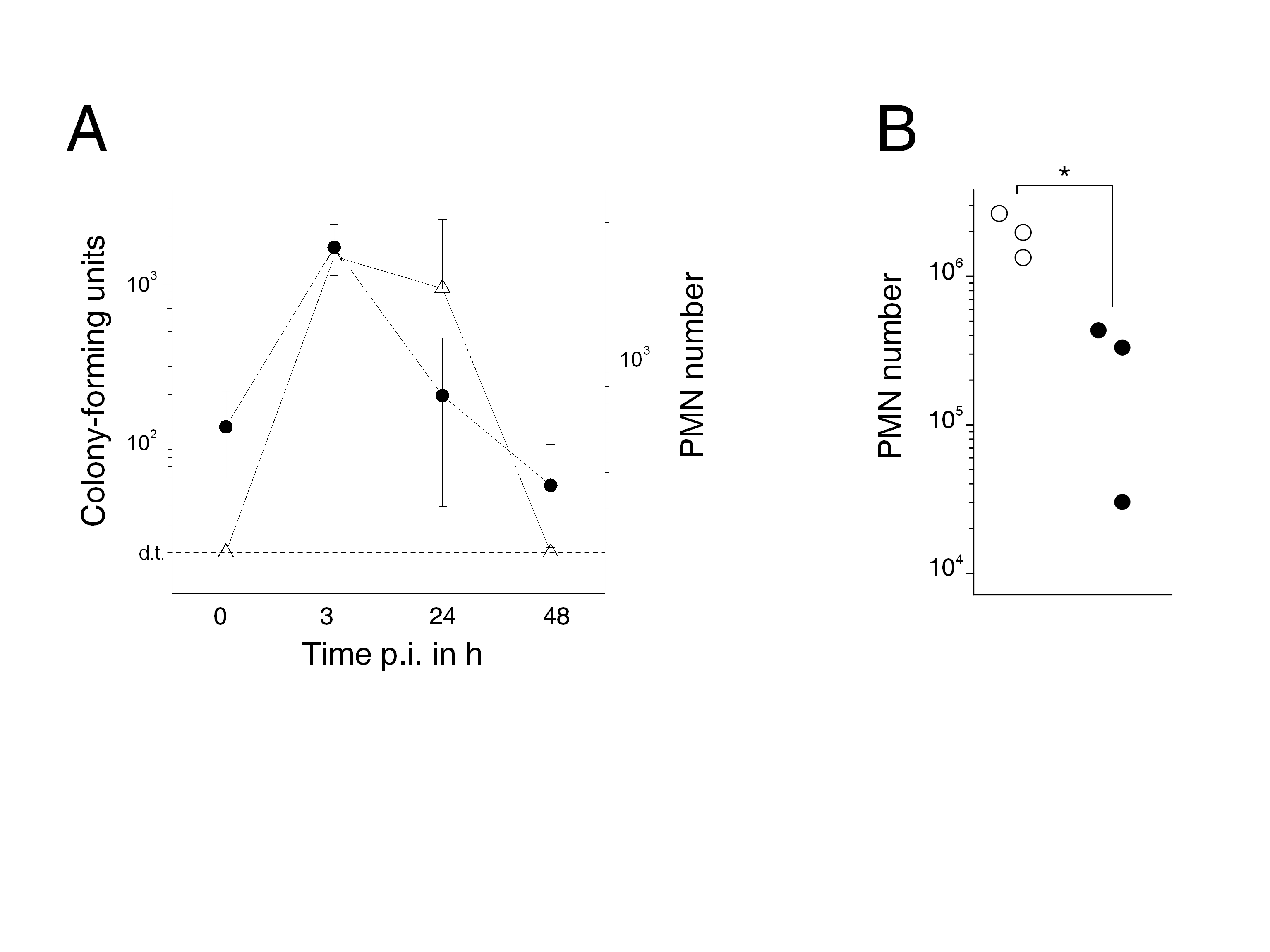

Supplement: Figure S4 — Neutrophil infiltration and colonization of Peyer’s patches after administration of a murine E. coli strain. A) Colonization levels of E. coli E2 (open triangles) and PMN infiltration (filled circles) in Peyer’s patches at various time intervals post infection (d.t., detection threshold for plating). The data might overestimate actual bacterial tissue loads due to potential contamination with residual luminal EcN especially at early time points after oral administration. Means and standard deviations are shown for groups of three mice. B) Neutrophil accumulation in Peyer’s patches two days after oral infection with pathogenic Salmonella in BALB/c mice. One day before Salmonella infection, mice received PBS (open circles), or a single dose of murine E. coli E2 (filled circles). Significance of differences between groups was determined using t-test (*, P<0.05). (TIF) [file pone.0035992.s004.tif]
